# Expression of MSP58 in human colorectal cancer and its correlation with prognosis

**DOI:** 10.1007/s12032-012-0284-y

**Published:** 2012-07-08

**Authors:** Hai Shi, Shu-Jun Li, Bo Zhang, He-Liang Liu, Chang-Sheng Chen

**Affiliations:** 1 State Key Laboratory of Cancer Biology, Department of Gastrointestinal Surgery, Xijing Hospital, The Fourth Military Medical University, Xi’an, 710032 Shanxi People’s Republic of China; 2 Department of Urology Surgery, Tangdu Hospital, The Fourth Military Medical University, Xi’an, 710032 Shanxi People’s Republic of China; 3 Department of Urology Surgery, Xijing Hospital, The Fourth Military Medical University, Xi’an, 710032 Shanxi People’s Republic of China; 4 Department of Health Statistics, The Fourth Military Medical University, Xi’an, 710032 Shanxi People’s Republic of China

**Keywords:** MSP58, Colorectal cancer, Prognosis, Immunohistochemistry, UICC

## Abstract

We had reported that MSP58 regulates colorectal cancer cell proliferation, development, and apoptosis, by the cyclin D1-cyclin-dependent kinase 4-p21 pathway. In this study, MSP58 protein expression was examined by immunohistochemistry in 499 specimens of CRC. The relationship between various clinicopathological features and overall patient survival rate was analyzed. The association of MSP58 expression with the 499 CRC patients’ survival rate was assessed by Kaplan–Meier and Cox regression. Using ROC curve to provide sensitivity and specificity of the score of MSP58 predicts local recurrence and survival of CRC patients. The expression of MSP58 was positively correlated with the depth of invasion (*P* < 0.001), local recurrence (*P* = 0.008), tumor grade (*P* = 0.002), and UICC stage (*P* < 0.001). The Kaplan–Meier survival analysis demonstrated that the survival time of CRC patients with low expression of MSP58 was longer than those with high expression during the 5-year follow-up period (*P* < 0.001). COX regression analysis indicated that high expression of MSP58 (*P* < 0.001), depth of invasion >pT_1_ (*P* = 0.008), distant organ metastasis (pM_1_) (*P* < 0.001), regional lymph node metastasis (≥pN_1_) (*P* < 0.001), and local recurrence (Yes) (*P* = 0.007) were independent, poor prognostic factors of CRC. ROC curve showed the score of MSP58 expression level did provide a maximal sensitivity and specificity to predict local recurrence and survival of CRC patients. Our results demonstrated MSP58 might serve as a novel prognostic marker that is independent of, and additive to, the UICC staging system.

## Introduction

Colorectal cancer (CRC) is the fourth most common malignant tumor in China and the fifth most frequent cause of cancer-related death [1, 2]. Despite curative surgical resection of the primary tumor and adjuvant chemotherapy, 40–50 % of the patients ultimately die of local recurrence and metastases [3, 4]. Tumor growth and metastasis result from a complex cascade of biological processes. Therefore, understanding key factors in these processes is crucial to the design of new treatment modalities. Although many molecular markers, including carcinoembryonic antigen (CEA), have been exploited for detecting CRC, these lack sensitivity and specificity for evaluating the prognosis of CRC patients [5–7]. Thus, there is an urgent demand for research into novel molecular markers that can serve as diagnostic and prognostic markers for CRC.

MSP58 was first identified as a nuclear protein interacting with the proliferation-related nucleus protein p120 [8]. The following studies showed that MSP58 could function in transcription regulation in the nucleus through interactions with transcription factors Daxx, STRA13, and also RNA-binding protein FMR [9, 10]. A study showed that TOJ3, the quail homologue of MSP58, displayed transformation activity in jun-transformed fibroblasts [11], whereas the tumor suppressor gene PTEN could suppress its transforming activity [12]. In addition, our previous studies demonstrated that MSP58 interacted with N-myc downstream-regulated gene 2 (NDRG2) in nucleus, which exerted important functions in cell differentiation and tumor proliferation [13]. Furthermore, we found that the expression of MSP58 was significantly up-regulated in high-grade glioblastoma and colorectal carcinoma tissues, and over-expression of MSP58 was involved in tumor growth, metastasis, cell cycle control, and invasion [14, 15]. We also reported that MSP58 regulates colorectal cancer cell proliferation, development, and apoptosis, by the cyclin D1-cyclin-dependent kinase 4-p21 pathway [15].

Nevertheless, there is lack of large sample of CRC patient to evaluate whether MSP58 can be served as a sensitive indicator to predict the prognosis of CRC patients. In the present study, we used immunohistochemistry to investigate MSP58 expression in 499 CRC patients and explored, for the first time, the possible relationship between MSP58 expression and prognosis in CRC.

## Methods

### Patients and specimens

This study was approved by the Ethics Committee of the Fourth Military Medical University. Fresh colorectal carcinoma specimens and patient-matched adjacent tissues were collected from 499 patients in the Department of Gastrointestinal Surgery of Xijing Hospital at the Fourth Military Medical University (Xi’an, China) between October 2000 and November 2003. Of the 499 patients, 40 (8.0 %, some CRC patients with stage IV of UICC) received neoadjuvant chemotherapy, 438 (87.8 %, CRC patients with stage IIB, IIC, III, and IV of UICC) underwent surgery alone and received subsequent chemotherapy, and 61 (12.2 %, CRC patients with stage I and IIA of UICC) only received surgical treatment. Histomorphology of all primary tumor specimens and regional lymph nodes was confirmed with hematoxylin–eosin staining according to the International Union against Cancer UICC classification. Cancer tissues, along with normal tissues that were at least 5 cm away from the cancer, were obtained from the patients. All specimens were fixed in 10 % formalin and embedded in paraffin, and 4-um serial sections were examined by immunohistochemistry.

The mean age of the 499 patients was 59 years (range: 21–84 years) with 191 women and 308 men. All 499 patients’ survival information of 71 months postoperative follow-up was received by telephone and mail. The median follow-up period was 41.2 months (range: 10–71 months). Patients’ characteristics, such as gender, age, location of the tumor, L stage, V stage, UICC stage, local recurrence, and tumor stage factors, were obtained from the medical records. Patient characteristics are summarized in Table 1. All resection samples were confirmed to be CRC by clinical pathology. All the patients were staged based on the UICC staging system. Of the 499 patients, 34 (6.8 %) had T1-stage, 80 (16.0 %) had T2-stage, 300 (60.2 %) had T3-stage, and 85 (17.0 %) had T4-stage CRC.

**Table 1:** Correlation between clinicopathological characteristics and Msp58 expression

	Msp58(∓)	Msp58(++)	Msp58(+++)	*P*
Age				0.184
<65	160(62.3)	43(16.7)	54(21.0)	
≥65	138(57.1)	41(16.9)	63(26.0)	
Gender				0.293
Male	181(58.8)	46(14.9)	81(26.3)	
Female	117(61.3)	38(19.9)	36(18.8)	
Location				0.197
Right colon	32(49.2)	14(21.6)	19(29.2)	
Left colon	61(62.2)	17(17.4)	20(20.4)	
Rectum	205(61.0)	53(15.8)	78(23.2)	
pT				<0.001
pT_1_ + pT_2_	83(72.8)	24(21.1)	7(6.1)	
pT_3_ + pT_4_	215(55.8)	60(15.6)	110(28.6)	
pN				0.613
pN_0_	82(62.1)	17(12.9)	33(25.0)	
pN_1_	150(18.5)	46(60.2)	53(21.3)	
pN_2_	66(55.9)	21(17.8)	31(26.3)	
pM				0.830
pM_0_	202(59.9)	58(17.2)	77(22.9)	
pM_1_	96(59.6)	25(15.5)	40(24.9)	
L stage				0.427
L_0_	160(61.1)	45(17.2)	57(21.7)	
L_1_	138(58.2)	39(16.5)	60(25.3)	
V stage				0.141
V_0_	165(63.0)	40(15.3)	57(21.7)	
V_1_	133(56.1)	44(18.6)	60(25.3)	
UICC				<0.001
I + II	181(79.0)	34(14.8)	14(6.2)	
III + IV	117(43.3)	50(18.5)	103(38.2)	
Tumor grade				0.002
Well	79(61.7)	23(18.0)	26(20.3)	
Moderately	187(63.8)	42(14.3)	64(21.9)	
Poorly	32(41.0)	19(24.4)	27(34.6)	
Local recurrence				0.008
Yes	81(68.6)	21(17.8)	16(13.6)	
No	217(57.0)	63(16.5)	101(26.5)	

### Antibodies and reagents

Rabbit anti-MSP58 was a gift from Dr Laetitia Davidovic [14–16]. Goat anti-rabbit immunoglobulin G (IgG) was purchased from Sigma-Aldrich. Rabbit SP immunostaining kit was from ZYMED (ZSGB; Beijing, China).

### Immunohistochemistry

Immunohistochemistry was performed using the avidin–biotin–peroxidase method on all 499 colorectal cancer tissue specimens. All sections were deparaffinized in xylene and dehydrated through a gradient concentration of alcohol before endogenous peroxidase activity was blocked with 0.5 % H_2_O_2_ in methanol for 10 min. Nonspecific binding was blocked by incubating sections with 10 % normal goat serum in phosphate-buffered saline (PBS) for 1 h at room temperature. Without washing, sections were incubated with rabbit anti-MSP58 (1:300) in PBS at 4 °C overnight in a moist box. Negative controls were performed by replacing the primary antibody with pre-immune rabbit serum. Biotinylated goat anti-rabbit IgG (1:400, Sigma) was incubated with the sections for 1 h at room temperature and detected with streptavidin–peroxidase complex. The brown color indication of peroxidase activity was obtained by incubating with 0.1 % 3,3-diaminobenzidine (Sigma) in PBS with 0.05 % H_2_O_2_ for 5 min at room temperature.

### Staining evaluation

For MSP58 staining, tissue specimens were examined separately by two pathologists under double-blinded conditions without prior knowledge of the clinical status of the specimens. Any disagreement was resolved by consensus after joint review. MSP58 expression in colorectal cancer was evaluated by scanning the entire tissue specimen under low magnification (×40) and then confirmed under high magnification (×200). An immunoreactivity score (IRS) system was applied. Expression of MSP58 was evaluated as the percentage of positive cells and staining intensity as previously described. The percentage of positive cells was evaluated quantitatively and scored as 0 for staining of ≤1 % of total cells counted, 1 for staining of 2–25 %, 2 for staining of 26–50 %, 3 for staining of 51–75 %, and 4 for staining of >75 % of the cells examined. Intensity was graded as follows: 0, no signal; 1, weak; 2, moderate; and 3, strong staining. A total “staining score” of 0–12 was calculated and graded as negative (−, score 0–1), weak (+, score 2–4), moderate (++, score 5–8), or strong (+++, score 9–12) [15, 17, 18].

### Statistical analysis

The relationship between MSP58 expression levels and clinicopathological factors was analyzed using the Wilcoxon–Mann–Whitney test. The overall survival time of CRC patients was defined as the time from the surgery to death due to cancer. The Kaplan–Meier method was used to determine the cumulative probability of survival, and data were analyzed with the log-rank test. Univariate and multivariate statistical analyses were done using the Cox regression model to investigate the effects of patients’ characteristics (MSP58 expression status, gender, age, location, extent of primary tumor, nodal status, metastasis, L stage, V stage, UICC stage, and tumor grade) on overall survival. A score was assigned to each variable for the Cox regression analysis. A value of *P* < 0.05 was considered statistically significant [15, 17, 18].

Additionally, receiver operator characteristic (ROC) curves were constructed for the expression of MSP58 as a predictor for “patient death” and “cancer local recurrence.” The ROC curve determines how effective a predicted risk value is at discriminating a bivariable outcome (in this case “death” or “no death,” or “local recurrence” or “non-local recurrence”) by constructing a graph with sensitivity on the *Y*-axis and one specificity on the *X*-axis. It depicts the inverse relationship between sensitivity and specificity [17].

## Results

### MSP58 immunohistochemistry

In previous study, we observed, qualitatively, that MSP58 was predominantly located in the nucleus and cytoplasm of CRC cells. From the subcellular localization of MSP58 in CRC cell lines, we concluded that MSP58 lies predominantly in the nucleus, with a small fraction detectable in the cytoplasm [15]. In the present study, we found by average staining score that positive expression of MSP58 in CRC cells was more frequently than adjacent non-cancerous tissue cells, and the expression of MSP58 protein in highly differentiated tumor tissues was lower than in moderately or poorly differentiated CRC tissues.

### Relationship between MSP58 expression and clinicopathological characteristics

The correlation between the clinicopathological characteristics and MSP58 expression is shown in Table 1. According to the immunohistochemical results, 298 (59.7 %) of the 499 colorectal cancer samples were categorized as exhibiting negative or weak staining (∓). In contrast, 84 (16.8 %) and 117 (23.5 %) were scored as exhibiting moderate positive staining (++) and strong positive staining (+++), respectively. MSP58 expression was correlated with the depth of invasion (*P* < 0.001), local recurrence (*P* = 0.008), tumor grade (*P* = 0.002), and UICC stage (*P* < 0.001). However, MSP58 expression was not correlated with patient age, gender, tumor location, L stage, V stage, regional lymph node metastasis, or distant metastasis (see Table 1).

### Survival analysis

**Fig. 1:**
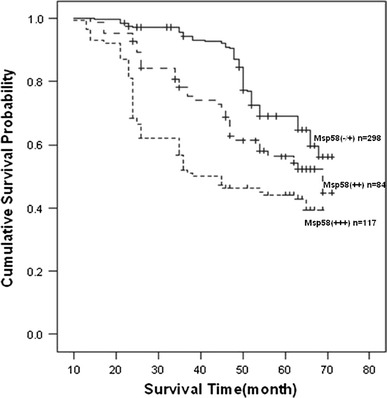
Overall survival of patients determined by the immunoreactivity of MSP58. Overall survival analysis using the Kaplan–Meyer method revealed that CRC patients with relatively low expression of MSP58 had a more favorable prognosis compared to those with high expression (*P* < 0.001)

The mean patient follow-up time was 71 months with a median value of 62.8 months. The 5-year survival rate of 499 patients was 62.5 %. The overall survival analysis using the Kaplan–Meyer method revealed that the prognosis of CRC patients with no or weak MSP58 expression was significantly better than those with high or moderate MSP58 expression, and moderate expression was better than high expression (see Fig. 1; *P* < 0.001).

**Fig. 2:**
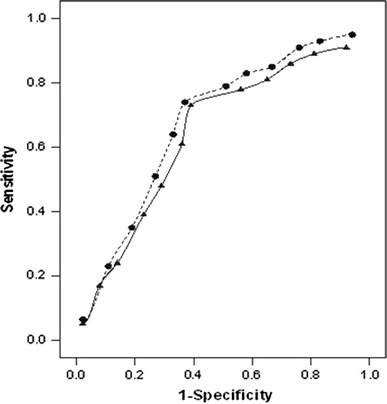
Receiver operating characteristic (ROC) curve for local recurrence (*circles*) within 5 years and survival (*triangles*) after 5 years

The ROC curve for recurrence within 71 months (see Fig. 2) showed that the score 7 of MSP58 expression level provided maximal sensitivity (0.64) and specificity (0.77). Similarly, the curve for survival after 71 months (see Fig. 2) showed that the score 6 of MSP58 expression level provided a maximal sensitivity (0.62) and specificity (0.75). The area under the curve for recurrence was 0.78 and for survival was 0.76. This shows that the score of MSP58 expression level did predict local recurrence and survival.

Both univariate and multivariate analyses showed that depth of invasion (pT3 or pT4), regional lymph node metastasis (≥pN_1_), distant tumor metastasis (pM_1_), local recurrence (yes), and high-expression MSP58 (+++) were independent, poor prognostic factors of CRC. However, age (≥65 years), gender (male), location (rectum or sigmoid), L stage (L_1_), V stage (V_1_), and tumor grade (≥G2) were not related to the prognosis of CRC (see Tables 2, 3).

**Table 2:** Cox univariate analysis

Variables	Wald Chi-square	*df*	*P*
Age	2.260	1	0.133
Gender	2.709	1	0.100
Location	4.163	1	0.041
pT	8.782	1	0.003
pN	28.716	1	<0.001
pM	84.258	1	<0.001
L Stage	1.746	1	0.186
V stage	1.814	1	0.178
Tumor grade	0.281	1	0.596
UICC	24.884	1	<0.001
Msp58	133.800	1	<0.001
Local recurrence	12.508	1	<0.001

**Table 3:** Cox multivariate analysis

Variables	Risk ratio (95 % confidence interval)	*P*
Age (≥65 years)	1.364 (0.869–2.142)	0.177
Gender (female)	1.076 (0.690–1.678)	0.748
Location (rectum or left colon)	1.072 (0.688–1.672)	0.758
Primary tumor (pT_3_ or pT_4_)	1.484 (1.109–1.986)	0.008
Regional lymph node metastasis (pN_1_ or pN_2_)	1.866 (1.400–2.487)	<0.001
Distant metastasis (pM_1_)	2.516 (1.791–3.535)	<0.001
L stage (L_1_)	0.985 (0.703–1.380)	0.930
V stage (V_1_)	1.422 (0.902–2.241)	0.129
Tumor grade (G2 or G3)	0.795 (0.564–1.121)	0.190
UICC (III or IV)	0.675 (0.463–0.985)	0.042
Msp58 (++ or +++)	5.419 (3.795–7.739)	<0.001
Local recurrence (yes)	0.644 (0.466–0.889)	0.007

## Discussion

Accurately predict CRC patients’ fate will be very helpful to determine proper adjuvant therapy. Currently, one relies heavily on traditional pathologic variables, such as tumor size, lymph node status, and tumor grade. UICC stage of tumor is the gold standard for determining prognosis in patients with colorectal cancer. But, patients with similar stages of disease may have wide discrepancy in survival [19–23]. Several new molecular prognostic factors, such as p53 and K-RAS mutations, are being evaluated in the hope that they may contribute to better assessment of survival probability [24–26]. However, they are still unlikely to accurately predict the probability of cancer recurrence following surgery, and consequently, it is difficult to apply tailored treatment for each individual patient. Therefore, it will be very urgent to find novel, reliable molecular markers that can serve as early diagnostic and prognostic markers for CRC.

Nucleolar MSP58 was initially identified through its interaction with the proliferation-related nucleolar protein p120, and its overexpression leads to enlargement of nucleoli. MSP58 has also been reported to interact with several other proteins. It is reported to play a role in modulation of Daxx-dependent transcriptional repression and is associated with the transcriptional activity of the basic-helix-loop helix (bHLH) transcription factor stimulated by retinoic acid 13 (STRA 13). MSP58 is reported to interact with Mi-2b in the nucleolus and to up-regulate ribosomal gene transcription [27–29].

Recently, MSP58 was shown to behave as a new oncogene and its transformation activity was inhibited by physical interaction with the PTEN tumor suppressor. In previous studies, we identified MSP58 as a novel Ndrg2-interacting protein and found that Ndrg2 could alter the role of MSP58 in cell proliferation [30]. It had been proved that Ndrg2 served as a tumor suppressor gene involved in gastric cancer [31], colorectal cancer [32], hepatocellular carcinoma [33], esophageal squamous cell carcinoma [18], and thyroid cancer [34]. Our group reported that there was aberrant expression of MSP58 has been found in human glioma, and RNAi-mediated inhibition of MSP58 decreases tumor growth, migration, and invasion in a human glioma cell line. We also found that MSP58 regulates colorectal cancer cell proliferation, development, and apoptosis, by the cyclin D1-cyclin-dependent kinase 4-p21 pathway. These findings suggested that MSP58 gene may serve as a new oncogene involved in some human cancer process.

In this study, we included a group of 499 CRC patients who underwent curative resection. An advantage of using immunohistochemistry is that it is relatively cheap and readily amenable to standardization in terms of methodology and interpretation, making it applicable for routine clinical use. In accordance with recent revisions on prognostic factors, our uni and multivariate analysis confirmed a significant level of MSP58 in this cohort of colorectal cancer, but no or weak expression in normal epithelial cells.

MSP58 was highly expressed with depth of invasion, especially in T4 and T3 carcinomas but no or weak expression in T2 and T1 carcinomas (*P* < 0.001). Therefore, depth of invasion is a very important prognostic factor, suggesting that the patients selected in this retrospective study were representative of colorectal cancer and similar to those evaluated in other prognostic studies. Nevertheless, depth of invasion could not be an independent prognostic factor since it is related to UICC stage.

Another important finding in our study was that local recurrence was detected more frequently in MSP58-positive tumors as compared with MSP58-negative cases (*P* = 0.008), and ROC curve also shown that the score of MSP58 expression level did provide a maximal sensitivity and specificity to predict local recurrence and survival of CRC patients. This finding also corroborated that our previous report that MSP58 involved in CRC cell cycle regulation. The same tendency was observed in UICC stage (*P* < 0.001) and tumor stage (*P* = 0.002). However, MSP58 expression was not correlated with patient age, gender, tumor location, L stage, V stage, regional lymph node metastasis, or distant metastasis.

Consistently, the overall survival rate tended to be lower for patients with MSP58-positive tumors than those with MSP58-negative tumors (*P* < 0.001). Kaplan–Meier analysis of the survival curves showed a significantly worse overall survival rate for patients whose tumors had high MSP58 levels, indicating that high MSP58 protein level is a marker of poor prognosis for patients with colorectal cancer. The Cox proportional hazards model adjusted for age, gender, tumor status, and UICC stage showed the same trend as the Kaplan–Meier postoperative survival curve. Moreover, multivariate analysis showed MSP58 expression to be an indicator of worse outcome, independent of the known clinical prognostic indicators such as UICC stage. These data suggest that MSP58 expression was correlated with worse outcome and might be an independent prognostic factor for patients with colorectal cancer. It could constitute a useful prognostic marker additive to the UICC staging system for these patients, who are more likely to have disease recurrence and are thus good candidates to receive aggressive adjuvant chemotherapeutic treatment.

Our work provides convincing evidence that MSP58 expression of collected colorectal cancer specimens is closely correlated with poor overall survival rate and might be a novel, reliable prognostic marker for colorectal cancer. It can be used as an adjunct to the UICC stage system to improve prognostication for an individual patient, particularly in the first 5 years after diagnosis of colorectal cancer.

## Abbreviations

MSP58: 58-kDa micro-spherule protein
CRC: Colorectal cancer
ROC: Receiver operator characteristic
CEA: Carcinoembryonic antigen
Daxx: Death domain-associated protein
STRA 13: Stimulated by retinoic acid 13
PTEN: Phosphatase and tensin homology deleted on chromosome ten
NDRG2: N-myc downstream-regulated gene 2
bHLH: Basic-helix-loop helix
IRS: Immunoreactivity score
PBS: Phosphate-buffered saline
